# Evaluation of sexual dysfunction in gynecologic cancer survivors using DSM-5 diagnostic criteria

**DOI:** 10.1186/s12905-021-01559-z

**Published:** 2022-01-05

**Authors:** Hao Lin, Hung-Chun Fu, Chen-Hsuan Wu, Yi-Jen Tsai, Yin-Jou Chou, Chun-Ming Shih, Yu-Che Ou

**Affiliations:** 1 Department of Obstetrics and Gynecology, Kaohsiung Chang Gung Memorial Hospital and Chang Gung University College of Medicine, Kaohsiung, Taiwan, ROC; 2 Department of Obstetrics and Gynecology, Chiayi Chang Gung Memorial Hospital, Chiayi, Taiwan, ROC; 3 Graduate School of Human Sexuality, Shu-Te University, Kaohsiung, Taiwan, ROC

**Keywords:** Gynecologic cancer survivors, Sexual dysfunction, DSM-5

## Abstract

**Background:**

In gynecologic cancer survivors, female sexual dysfunction (FSD) remains under-investigated. We attempted to estimate the prevalence of FSD associated with distress in gynecologic cancer survivors using diagnostic and statistical manual of mental disorders fifth edition (DSM-5) diagnostic criteria and to identify women at risk for FSD.

**Methods:**

We conducted a cross-sectional analysis of premenopausal women aged 20–50 with various gynecologic cancers at least one year after treatment between January 2017 and December 2019. Data of sociodemographics and physical conditions were collected via face-to-face interview during outpatient clinic visits. The domains we used to define FSD were based on DSM-5 diagnostic criteria. Statistical analysis was carried out using Student's t test, Chi-square test and multiple logistic regression.

**Results:**

A total of 126 gynecologic cancer survivors with a mean age of 42.4 years were included for analysis and 55 of them (43.7%) were diagnosed as having FSD associated with distress based on DSM-5 criteria. More than half of women (65.1%) reported decreased sexual satisfaction after cancer treatment. According to DSM-5 definition, the most common female sexual disorders were sexual interest/arousal disorder (70.9%), followed by genitopelvic pain/penetration disorder (60.0%), and orgasmic disorder (20.0%). In multiple logistic regression model, endometrial cancer diagnosis was the only independent factor predicting less influence of cancer treatment on FSD (OR 0.370; 95% CI 0.160, 0.856).

**Conclusion:**

The first study to use DSM-5 criteria for estimation of FSD prevalence. This enables clinicians to identify which women are actually needed to seek medical help. A prevalence of 43.7% of FSD associated with distress was found in a group of gynecologic cancer survivors with the most common being sexual interest/arousal disorder. Endometrial cancer survivors were at low risk for developing FSD after treatment.

## Background

There is a steady increase in the number of cancer survivors in last three decades due to the advances in cancer diagnosis and treatment. Generally, there is currently an estimated of 55–60% 5-year overall survival rate of all cancers indicating a substantial number of population in survivorship [1, 2]. Furthermore, most of gynecologic cancer patients were diagnosed with stage I and II diseases, and their life expectancy after complete treatment was even longer [3]. Due to disease nature and location, gynecologic cancer survivors are at high risk of developing sexual dysfunction, infertility, and body image alteration owing to the treatment modality, treatment-related genital organ deformities, and hormonal changes. These may occur immediately following treatment and even during long-term follow up [4, 5]. Recently, efforts have focused on how to identify this population and to improve quality of life of survivors. However, sexual health is still an under-researched area in gynecologic oncology and rarely discussed with care providers due to lack of knowledge.

Traditionally, female sexual dysfunction (FSD) refers to problems during any phase of the sexual response cycle, and also includes dyspareunia during sexual activity. The prevalence of FSD in healthy women varied, ranging from 26 to 60% according to different publications [5–8]. The large discrepancy is mainly due to different classification system of FSD and the complexity of female sexual response when compared with that of males. Furthermore, most epidemiologic definitions of FSD refer to sexual problems without requiring distress to be present. With the more understanding of female sexual response cycle, the new definition of FSD in the diagnostic and statistical manual of mental disorders-fifth edition (DSM-5) has addressed the importance of psychosocial factors that drive sexual response [9]. Furthermore, sexual dysfunctions in the DSM-5 now require a period of approximately 6 months of symptoms to meet the criteria for diagnosis. The DSM-5 also includes the requirement of experiencing the disorder 75–100% of the time to reduce over-diagnosis of sexual dysfunctions. In addition, the disorder must be deemed to have caused significant distress. The spirit of DSM-5 enables clinicians to identify which women are actually needed to seek medical help. Only few published studies have measured FSD associated with personal distress. In a United States national survey of healthy women aged 18 years and older, the authors found a prevalence of 43% of any sexual problem but dropped to 12% if personal distress was present [10]. To date, there is no data of estimated prevalence of FSD combined with distress in gynecologic cancer survivors.

The current study is a cross-sectional survey of gynecologic cancer survivors in a single institute. The purpose was to estimate the prevalence of FSD associated with personal distress using DSM-5 diagnostic criteria, and to identify at-risk subgroups who may actually benefit from early intervention.

## Methods

### Study design and patients’ selection

This cross-sectional study was conducted between January 2017 and December 2019 with patients at the gynecologic oncology outpatient unit in Kaohsiung Chang Gung Memorial Hospital, Taiwan, and has been approved by the Institutional Review Board of our hospital. All participants had received various treatments at least one year ago for their gynecologic cancers at the time of the inclusion. The inclusion criteria of study were as follows: being 20–50 years and premenopausal status before treatment, having a partner, being in the disease-free period, having had sexual attempts within one year, and being willing to participate in the study. Women who were pregnant, lactation, smoking, alcoholism, or drug-abuser were excluded.

### Outcome measures

All eligible patients were offered participation in the survey during routine outpatient clinic visits. After informed consent, eligible women were instructed to complete the survey form in a private room located in the outpatient unit. A well-trained clinical research associate (YCT) checked the survey form for completion and provided assistance replying questions if needed. After completion, all participants were awarded a gift valued 100 New Taiwan Dollars. Completed survey forms were not linked with identifying information.

The survey form consisted of socio-demographic characteristics of patients, diseases nature and its treatment, physical/medical condition, drug history of participants/partners that would probably affect sexual function, and sexual frequency/satisfaction after cancer treatment. The female sexual functioning was accessed by attending physicians (HL, HCF, YCO) via face-to-face interview once the patients were willing to participate the study. The participants’ partners were allowed to be at the scene during interview. Briefly, women were diagnosed as having FSD if at least any one of the three disorders (sexual interest/arousal disorder, orgasmic disorder, and genitopelvic pain/penetration disorder) was present based on DSM-5 diagnostic criteria. More importantly, a woman must experience the symptoms for at least six months and suffer "significant distress" to meet the diagnosis. The diagnosis of sexual interest/arousal disorder must include three or more of the following items (1) little interest in sex, (2) few thoughts related to sex, (3) decreased start and rejecting of sex, (4) little pleasure during sex most of the time (75–100%), (5) decreased interest in sex even when exposed to erotic stimuli, (6) little genital sensations during sex most of the time (75–100%). The diagnosis of orgasmic disorder was made if a woman cannot achieve orgasm despite adequate stimulation. The diagnosis of genitopelvic pain/penetration disorder must include two or more of the following items (1) multiple episodes of difficulty with vaginal penetration, (2) pain associated with intercourse attempts, (3) anticipation of pain due to attempted intercourse, (4) tensing of the pelvis in response to attempted penetration.

### Statistical analysis

Data were analyzed using SPSS software (Version 22; IBM Institute, Cary, NC, USA). Percentage distributions were used to analyze the socio-demographic and descriptive characteristics of the patients; means and standard deviations were used to calculate the continuous variables. In order to analyze the relation between the variables, Student t test, Chi-square test, and One-way ANOVA tests were used. In determining the independent factors predicting sexual dysfunction, a multivariate logistic regression analysis was used. A *p* value less than 0.05 was considered as statistically significant.

## Results

### Participants characteristics

A total of 201 patients were screened and approached for the study. Sixty-four patients rejected our invitation due to time constrains or privacy matter. Eleven were not eligible due to evidence of recurrent disease, currently being on active treatment, or heavy smoker. Finally, 126 participants were enrolled for this study. Table 1 lists summary statistics for demographic and clinical characteristics for the 126 participants. Mean age was 42.4 years; median time from diagnosis was 4.9 years. Almost all the women reported being married or in a stable relationship (99%). Cervical, ovarian, and endometrial cancer diagnoses composed 95.2% of the cancer diagnoses. Subjects received a variety of treatments and treatment combinations, including surgery, radiation, chemotherapy, and hormone therapy. The majority of gynecologic cancer survivors (88.9%) were International Federation of Gynecology and Obstetrics (FIGO) stage I and II diseases. Only six women (1.6%) had recurrent history but all of them were free of disease at the time of inclusion. Potential medical diseases that would probably affect sexual function were found in 23% of the participants and 18.3% of their sexual partners (most were diabetes or cardiovascular diseases, none had anxiety or depression disorder). Forty-seven women (37.3%) had current medication for at least one month, most were for treatment of hypertension.

**Table 1:** Demographic characteristics of 126 gynecologic cancer survivors

	n (%)		n (%)
Age, year (mean ± SD)	42.4** ± **6.29	Sexual partner	
Birth place		Single	125 (99.2)
Taiwan	120 (95.2)	Multiple	1 (0.8)
China	4 (3.2)	Cancer type	
Others	2 (1.6)	Endometrium*	37 (29.4)
Education		Ovary*	41 (32.5)
Junior high school or less	24 (19.0)	Cervix	45 (35.7)
Senior high school	39 (31.0)	Others	6 (4.8)
College or University	61 (48.4)	Stage (FIGO)	
Graduate School	2 (1.6)	I	93 (73.8)
Residence		II	19 (15.1)
Village	11 (8.7)	III	9 (7.1)
Township	4 (3.2)	IV	5 (4.0)
County	41 (32.5)	Treatment	
City	70 (55.6)	Hysterectomy	89 (70.6)
Religion		Oophorectomy	42 (33.3)
Buddhist or Taoism	84 (66.7)	Radiotherapy	28 (22.2)
Christian or Catholicism	11 (8.7)	Chemotherapy	55 (43.7)
Not specified	31 (24.6)	Recurrent history	6 (1.6)
Marital status		Hormone replacement	22 (17.5)
Unmarried	14 (11.1)	Self medical disease	29 (23.0)
Married or cohabiting	104 (82.5)	Partner medical disease	23 (18.3)
Separate or divorced	8 (6.4)	Current medication	47 (37.3)

*FIGO* International Federation of Gynecology and Obstetrics, *SD* Standard deviation

*Three patients had synchronous endometrial and ovarian cancer

### Sexual functioning of the participants

Table 2 lists the sexual frequency and satisfaction of participants after cancer treatments. Thirteen women (10.4%) reported failed sexual attempts while 82 women (65.1%) reported decreased sex frequency as compared to pre-treatment. One hundred and two women (81.6%) had sex frequency being reported as one to three times a month or less. Decreased sex satisfaction was also noted in 52.2% of women who had sex activity. We also evaluated potential reasons of these sex problems and found that the five common causes in descending order were vagina pain (33.9%), fatigue (25.6%), aging (24.8%), poor general health (24.0%), and low abdomen discomfort (23.1%). Interesting, 19% of women reported fear of cancer recurrence as having sex and about 18% reported sex problems related to their partners. Detailed reasons for sex problems are listed in Table 3. Overall, a prevalence of 43.7% (55 women) of FSD was found in a group of gynecologic cancer survivors. Of the 55 women, 18 (32.7%) of them reported as having moderate to severe distress, 39 (70.9%) had sexual interest/arousal disorder, 33 (60.0%) had genitopelvic pain/penetration disorder, and 11 (20.0%) had orgasmic disorder (Table 4). Twenty-four (43.6%) women had more than one disorders.

**Table 2:** Sex frequency and satisfaction of the participants after cancer treatment

	N = 125* n (%)		N = 125* n (%)
Sex frequency		Sex frequency	
Never after cancer treatment	13 (10.4)	1–3/month	56 (44.8)
1–5/year	20 (16.0)	1 or more/week	23 (18.4)
6–11/year	13 (10.4)		

	N = 126 n (%)		N = 113# n (%)
Sex frequency		Sex satisfaction	
Decreased	82 (65.1)	Decreased	59 (52.2)
Similar	41 (32.8)	Similar	52 (46.0)
Increased	3 (2.4)	Increased	2 (1.8)

*One participant elective to skip to answer this question

^#^Exclude those who had no sex after cancer treatment

**Table 3:** Reasons for sex problems of 126 gynecologic cancer survivors

Reasons of sex problems	n (%)	Reasons of sex problems	n (%)
Aging	30 (24.8)	Lack of privacy	8 (6.6)
Cancer treatments	22 (18.2)	Sex unsatisfaction	7 (5.8)
Fear of recurrence	23 (19.0)	Currently no sexual partner	0 (0.0)
Poor health	29 (24.0)	Partner sex dysfunction	2 (1.7)
Poor body image	3 (2.5)	Partner poor skills	1 (0.8)
Should not have sex	7 (5.8)	Partner rejection	9 (7.4)
Low abdomen discomfort	28 (23.1)	Partner sex unsatisfaction	2 (1.7)
Vaginal pain	41 (33.9)	Poor partner relationship	8 (6.6)
Fatigue	31 (25.6)	Others	6 (5.0)

n > 126 due to presence of multiple reasons

**Table 4:** Fifty-five gynecologic cancer survivors with disorders of female sexual dysfunction based on DSM-5 diagnostic criteria

Disorder	n (%)*
Sexual interest/arousal disorder	39 (70.9)
Orgasmic disorder	11 (20.0)
Genitopelvic pain/penetration disorder	33 (60.0)

*n > 55 due to presence of multiple disorders in 24 women

### Clinical factors predicting FSD

Univariate analysis showed that endometrial cancer (*p* = 0.015) and hormone replacement (*p* = 0.037) were significant factors associated with FSD (Table 5). However, after adjusted for ovarian cancer diagnosis, chemotherapy, and hormone replacement, endometrial cancer was the only significant independent factor predicting no influence on FSD (odds ration 0.370; 95% confidence interval: 0.160, 0.856) (Table 6). We attempted to evaluate the differences of treatment between endometrial and non-endometrial cancer patients. We found that women with endometrial cancer had significant lower rate of radiation (2.7% vs. 31.8%, *p* < 0.001) and chemotherapy (18.9% vs. 54.5%, *p* < 0.001), but higher rate of oophorectomy (54.1% vs. 25%, *p* = 0.002), and similar rate of hysterectomy (81.1% vs. 67%, *p* = 0.114).

**Table 5:** Comparing women with and those without sexual dysfunction based on DSM-5 criteria (N = 126)

Parameters	Female sexual dysfunction (DSM-5)		*p* value
	Yes (n = 55)	No (n = 71)	
Age, year (mean ± SD)	42.33 ± 5.86	42.52 ± 6.65	0.872
Education			
Senior high school or less	24 (43.6%)	39 (54.9%)	0.209
College/University or above	31 (56.4%)	32 (45.1%)	
Residence			
Suburb	23 (41.8%)	33 (46.4%)	0.456
City	32 (58.2%)	38 (53.6%)	
Marital status			
Married	46 (83.6%)	58 (81.7%)	0.775
Unmarried/divorced	9 (16.4%)	13 (18.3%)	
Cancer type			
Endometrium*	10 (18.2%)	27 (38.0%)	0.015
Ovary*	23 (41.8%)	18 (25.4%)	0.051
Cervix	20 (36.4%)	25 (35.2%)	0.893
Others	3 (5.5%)	3 (4.2%)	0.748
FIGO stage			
I	43 (78.2%)	50 (90.9%)	0.370
II–IV	12 (21.8%)	21 (9.1%)	
Treatment			
Hysterectomy	41 (74.5%)	48 (67.6%)	0.309
Oophorectomy	21 (38.2%)	21 (29.6%)	0.275
Radiation	15 (27.3%)	13 (18.3%)	0.182
Chemotherapy	29 (52.7%)	26 (36.6%)	0.057
Time from treatment			
≤ 3 years	36 (65.5%)	36 (50.7%)	0.115
> 3 years	19 (34.5%)	35 (49.2%)	
Hormone replacement	14 (25.5%)	8 (11.3%)	0.037
Self medical disease	13 (23.6%)	16 (22.9%)	0.981
Partner medical disease	13 (23.6%)	10 (14.5%)	0.160
Current medication	22 (40.0%)	25 (35.7%)	0.623
Recurrent history	4 (7.3%)	2 (2.8%)	0.249

*FIGO* International Federation of Gynecology and Obstetrics, *SD* Standard deviation

^*^Three patients had synchronous endometrial and ovarian cancer

**Table 6:** Multivariate analysis for factors predicting female sexual dysfunction based on DSM-5 criteria

Parameters*	OR (95% confidence interval)	*p* value
Endometrial cancer	0.370 (0.160, 0.856)	0.020
Ovarian cancer	1.569 (0.685, 3.593)	0.287
Chemotherapy	1.433 (0.652, 3.148)	0.371
Hormone replacement	2.082 (0.768, 5.645)	0.149

*OR* Odds ratio

*Only for those with significant or marginal significant parameters in univariate analysis were included for multivariate analysis

## Discussion

In present study, we are the first of using DSM-5 criteria to investigate FSD associated with distress and found a prevalence of 43.7% in a group of gynecologic cancer survivors with the most common problem being sexual interest/arousal disorder. The prevalence rate was much higher than healthy women reported from previous studies although the assessment tools were different. In addition, we found that almost all treatment modalities involved in gynecologic oncology were associated with unfavorable sexual problems. Only for endometrial cancer survivors were at low risk of developing sexual dysfunction.

As we have mentioned previously, the prevalence of FSD varied due to different classification exist and complexity of female sexual response that make the diagnosis difficulty. Several instruments have been explored and the most widely used assessment tool was FSFI (female sexual function index) scoring system [11]. It was developed for the specific purpose of assessing domains of sexual functioning (e.g. sexual arousal, orgasm, satisfaction, pain) in clinical studies. Usually if a score less than a cutoff value (26.55), one may be classified as having FSD [12]. By using FSFI as assessment tool, Onujiogu et al. reported a prevalence of 89% FSD in low-risk endometrial cancer survivors without evidence of disease and one to five years out from primary surgical treatment alone [13]. Carter et al. reported a more even higher prevalence of 93.5% in female cancer survivors who had an FSFI score < 26.55 indicating sexual dysfunction, in which 36% of participants were gynecologic origin [14]. However, they only evaluated patients who were referred to sexual medicine department for sexual health issues management. Furthermore, almost half of study patients were still under active treatment. The relative high prevalence rate was most probably due to the fact that FSFI questionnaire didn’t take severity and duration of symptoms and also personal distress into diagnostic consideration and also due to different patient selection criteria used. With the current understanding of the complexities related to female sexual response cycle has prompted recommendation of a new classification system based on DSM-5 diagnostic criteria. The changes aim at increasing its validity and clinical usefulness. This development corrects what was seen as a flaw in previous sexual dysfunction diagnostic criteria, which did not have duration requirements [15]. In this study we found only 43.7% prevalent rate of FSD in gynecologic cancer survivors (27% in endometrial cancer, 56.1% in ovarian cancer, and 44.4% in cervical cancer), which was much lower than previous reports. Our results reflect the strict criteria of DSM-5, which could certainly avoid over-estimating the prevalence of FSD. Similar results were found in studies assessing healthy premenopausal women showed a prevalence of 40% but dropped to 12–25% if sexual problems associated with personal distress were taken into account [10, 16].

It has been reported that almost all types of treatment involved in gynecologic oncology affect female sexual functioning but we did not observe which one of these treatment modalities had more impact on sexuality than the others. Bilateral salpingo- oophorectomy is almost an inevitable surgical procedure for patients with ovarian and endometrial cancers. Physical changes after oophorectomy including loss of ovarian function, hot flashes, vaginal dryness, hair and skin changes, and mood changes. Tucker et al. reported a 80% of FSD in breast cancer survivors after risk reduction salpingo-oophorectomy (RRSO). Interesting, similar sexual outcome was noted after RRSO in women without breast cancer history [17]. These findings indicate the importance of sex hormone in maintaining sexual health. Simple hysterectomy itself does not negatively affect sexuality [18]. However, radical hysterectomy (RH) for early stage cervical cancer is associated with vaginal morbidity and bladder and bowel dysfunction [19]. In previous studies, RH significantly impaired females' sexual function regardless of surgical approach [20]. Radiation is also a common treatment modality for gynecologic cancers either as primary or adjuvant setting following surgery. It results in ovarian failure and long-term vaginal morbidity including stenosis, shortening, atrophy, fibrosis, and dyspareunia [21]. Almost all previous studies reported a persistent sexual dysfunction with limited improvement over time after radiotherapy for cervical cancer [22, 23]. Even for women receiving adjuvant brachytherapy alone, Damast et al. reported that 81% had FSFI score less than 26.55 suggesting FSD [24]. Chemotherapy plays an important role in the treatment of patients with gynecologic cancer as well. The systemic side effects of chemotherapy that aggravate a sense of a reduction in sexual attractiveness are fatigue, weight changes, insomnia, nausea/vomiting, fear, and anxiety. Domenici et al. investigated sexual function in ovarian cancer patients during chemotherapy [25]. They confirmed that ovarian cancer has a detrimental impact on intimacy especially in younger patients and during the first course of chemotherapy. However, the FSFI scores showed a moderate improvement 3 months later reflecting an importance of psychological effects on sexuality such as scared and rejection of the disease upon initial diagnosis. In present study even we investigated women who had completed her treatment for at least one year, we still found a 20–30% of participants reported psychological issues that would affect marital intimacy including fear of recurrence, should not have sex after cancer treatment, poor body image, partner rejection, and poor partner relationship. Psychological support might be required at any period after cancer diagnosis.

It is very interesting to discuss which of the gynecological cancer type would have the most possibility of developing sexual dysfunction after treatment. Only a few studies have examined regarding this issue. In a recent study of Guntupalli et al*.* they found that women who have an ovarian or cervical cancer diagnosis are at particularly high risk of sexual dysfunction [26]. The results agree with us that endometrial cancer diagnosis was at low risk for developing sexual dysfunction after treatment when compared with ovarian or cervical cancer, although FSD had been reported in 68–89% of endometrial cancer survivors [13, 27]. Majority of endometrial cancer is diagnosed and treated at an early stage due to early symptom thus adjuvant therapy following surgery is usually unnecessary. In our study, the rates of adjuvant radiotherapy and chemotherapy were significant lower in patients with endometrial cancer. Furthermore, there were 15 women with early stage disease received fertility preserving hormone therapy. All these findings could help explain why endometrial cancer diagnosis was a significant predictor for no sexual dysfunction after cancer treatment.

There are several limitations to present study. First, this study was a single-center design with a limited case number. Second, there was selection bias since a certain number of patients were excluded owing to personal reasons such as time constrains or privacy matter, although we assumed that these missing patients were at random. Third, face-to-face interviews might tend to lead a response bias towards denial of sexual problems, probably resulting in under-reporting of FSD prevalence. Fourth, absence of a control group (healthy women) may hamper our results. Further study with a high level of privacy design such as answering of questions online and inclusion of a control group should be conducted.

## Conclusion

The DSM-5 diagnostic criteria allowed first-line physicians to make the preliminary impression of FSD associated with distress very easily and to identify women actually needed for seeking medical help. Sexual issue is important to gynecologic cancer survivors, but is rarely discussed with clinicians owing to inadequate knowledge in this field. Oncology providers can ask about sexual concerns by putting emphasis on the severity and duration of symptoms in clinical practice. Personal distress can be assessed by just asking whether or not presence of frustration, grief, incompetence, sorrow, or worry. Patients at-risk such as cervical or ovarian cancer diagnosis can be routinely referred to specialists for more complex sexual issues monitoring and treatment.

## Data Availability

The datasets used and/or analyzed during the current study are available from the corresponding author upon reasonable request.

## Abbreviations

DSM-5: Diagnostic and statistical manual of mental disorders fifth edition
FIGO: International Federation of Gynecology and Obstetrics
FSD: Female sexual dysfunction
FSFI: Female sexual function index
RH: Radical hysterectomy
RRSO: Risk reduction salpingo-oophorectomy
